# Supplementary material for: Genomic and Phenotypic Variations Among Thai-53 and Mycobacterium leprae Clinical Isolates: Implications for Leprosy Pathogenesis and Research
Source: Pathogens. 2024 Nov 12;13(11):986. doi: 10.3390/pathogens13110986 (PMC11597610; doi:10.3390/pathogens13110986)
Supplement: Supplementary file 1 [file pathogens-13-00986-s001.zip › Supplementary Figure 1.pdf]

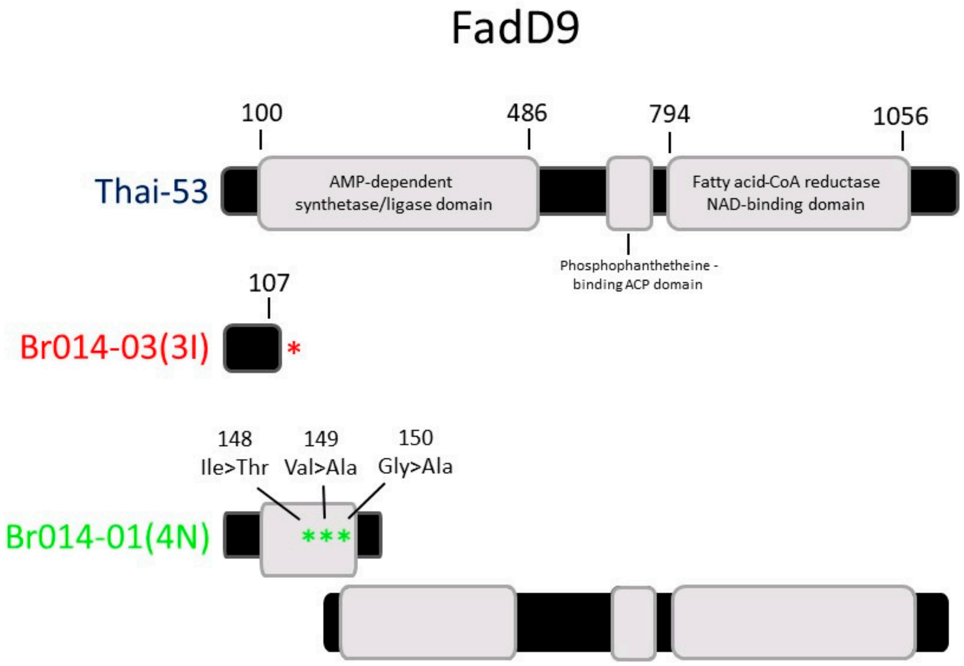

Supplementary Figure S1: SNPs and Indel impact over Acyl-CoA synthetase (FadD9) polypeptide chains in *M. leprae* Thai-53, Br014-03(3I) and Br014-01(4N). Br014-03(3I) present an SNP that introduces a stop codon, interrupting the protein at Gln 107 (red asterisk). The *fadD9* in Br014-01(4N) presents SNPs that result in aminoacid changes at positions 148, 149, and 150 within the putative AMP-binding domain (green asterisks), as well as an insertion-deletion that introduces a frameshift. Consequently, it possibly expresses two proteins: a 190-aminoacids protein (100% aminoacids identity from positions 1 to 147 in Thai-53) and a 1012-aminoacids proteins (100% aminoacids identity from positions 177 to 1188 in Thai-53).
